# Comparing the cross-national impact of the COVID-19 pandemic on care received by community-dwelling older adults in 2020 and 2021: restoring formal home care versus polarizing informal care?

**DOI:** 10.1007/s10433-024-00800-6

**Published:** 2024-01-24

**Authors:** Aviad Tur-Sinai, Netta Bentur, Paolo Fabbietti, Giovanni Lamura

**Affiliations:** 1 Department of Health Systems Management, The Max Stern Yezreel Valley College, 1930600 , Yezreel Valley, Israel; 2 School of Nursing, University of Rochester Medical Center, Rochester, NY 14642-8404 USA; 3 The Stanley Steyer School of Health Professions, Sackler Faculty of Medicine, Tel-Aviv University, Tel Aviv, Israel; 4 INRCA IRCCS—National Institute of Health and Science on Ageing, Unit of Geriatric Pharmacoepidemiology, 60124 Ancona, Italy; 5 INRCA IRCCS—National Institute of Health and Science on Ageing, Centre for Socio-Economic Research on Ageing, 60124 Ancona, Italy

**Keywords:** Informal care, Formal home care, COVID-19, Stringency Index, SHARE

## Abstract

The COVID-19 pandemic has changed the supply of formal and informal home care to older adults in many countries across the world. This study aims to compare the initial picture of how the supply of formal and informal home care to older adults in European countries and Israel changed during the first pandemic year (from mid-2020 to mid-2021) and to examine the changes that these countries made in the provision of adequate care to older adults. Using data from the two COVID-19 waves of SHARE, we show that the provision of formal home care services improved in the investigated period, as in 2021 the share of those who reported difficulties in receiving formal home care dropped significantly compared to the previous year. By contrast, informal care provision patterns experienced a growing polarization, with some countries continuing in reporting a strong support from this source, and others moving towards a remarkable reduction in the help coming from informal networks. These findings can serve as a basis for the development of evidence-based recommendations that can inform future care policies at the national level and to implement more sustainable models for older adults living in the community.

**Supplementary Information:**

The online version contains supplementary material available at 10.1007/s10433-024-00800-6.

## Introduction

The COVID-19 outbreak caught the world unprepared at the beginning of 2020 when, to tackle the surging pandemic, national governments started implementing a variety of public health and social containment measures. Most countries focused on the impact of the pandemic on the health of the general populations, and especially of those belonging to older age groups, but primarily who were already taken care of by residential care facilities (Rocard et al. 2021), while less attention was devoted to its effects on community-dwelling older adults (Miller et al. 2020). Therefore, although the vast majority of frail older adults live and are cared for at their home in the community, and many of them were already vulnerable before the outbreak, the preliminary responses to the epidemic overlooked this special population group as well as their family caregivers. Thus, the responses to their complex and sizable needs and to their requirement to an immediate attention by the public health system and by the welfare state in general were delayed, insufficient and unsatisfactory (Fraser et al. 2020; Cohen et al. 2020).

In a previous study aiming to identify the impact of the first wave of the COVID-19 pandemic on informal and formal care available to community-dwelling older adults (Tur-Sinai et al. 2021), we found a complex picture. While in the first months of the pandemic outbreak a significant proportion of older adults in a variety of European countries and Israel reported an increase in the amount of informal help provided by children, neighbours, friends and/or colleagues, some encountered great difficulties in receiving formal home care from professional bodies, due to the disruption affecting many formal care service providers (Burau et al. 2022). The situation varied greatly across countries during that time: in some, the amount of informal help grew, with no difficulties affecting formal care; in others, older people received less help from both formal and informal sources; and in a third group, older adults received less formal help and more informal help (Tur-Sinai et al. 2021).

These results impelled us to identify what picture could be drawn a year later, also in light of two crucial developments occurring during this period. Firstly, the health and welfare authorities in the countries started planning policy steps, developing services and properly addressing the deficiencies that may beset older adults. Secondly, widespread use of vaccination began at the end of the year 2020 (Polack et al. 2020; Baden et al. 2021), an intervention that not only led to lower rates of infections, hospitalizations, and deaths (Haas et al. 2021), but also enabled the lowering of the restrictions that dramatically had impacted the daily lives of older adults with care needs and their families, thus facilitating the formal services to get back to close-to-full operability (Dagan et al. 2021).

In light of the above, with this study we pursued two aims: firstly, to compare the initial picture of how the supply of formal and informal care to older adults in need in European countries and Israel changed during the first pandemic year (from mid-2020 to mid-2021); secondly, to examine the changes that these countries made in ensuring the provision of adequate care to older adults, based on both formal and informal services.

## Measures and methods

### Data source and study sample

This study draws on data collected by the Survey of Health, Aging and Retirement in Europe (SHARE), which seeks to better understand the dynamics of the growing population of persons aged 50 + and to provide a research infrastructure for public policymaking on behalf of the older-adult population. The data collected in SHARE make it possible to compare the health, economic situation, and welfare of older adults in 29 European countries over time by providing a multidisciplinary cross-national bank of microdata on health, psychological, and economic variables.

During the COVID-19 period, it became necessary to revise the way the SHARE data were collected. Data-gathering via interviews in respondents’ homes was halted and replaced by telephone questionnaires. The questionnaire used at the beginning of the collection process was discontinued in favour of one that gathers focused data about the way people aged 50 + were coping with the crisis. This special-purpose survey centred on a series of topics related to respondents’ general health, mental health, ways of coping with the virus in the medical sense, coping in the labour market, and social-network characteristics. The special-purpose survey was conducted twice during the COVID-19 period: first, among 52,000 people in twenty-eight European countries (including Israel) over a two-month period from June to August 2020 (hereinafter—Wave 1-COVID-19); second, among 51,000 people in twenty-eight European countries (including Israel) over a two-month period from June to August 2021 (hereinafter—Wave 2-COVID-19).

### Variables included in the analysis

To study the impact of the pandemic on the supply of care to older adults in need, we examined informal and formal home care separately, starting with the former due to its predominance in care and its crucial role in supporting older adults in the community.

In regard to informal care, older adults in Wave 1-COVID-19 period were asked whether, during the outbreak of the pandemic, they had received informal help outside of home. The question was: “*Since the outbreak of Corona, were you helped by others from outside of home to obtain necessities, e.g. food, medications or emergency household repairs*?” with “*Yes*” and “*No*” as possible answers. To assess the total extent of informal care that older adults received, the participants were asked whether, during the outbreak of the pandemic, they had received less, the same, or more informal help from their children, other relatives, or non-relatives such as neighbours, friends, or work colleagues. An average level of help received from these three sources (children, other relatives, and non-relatives) was calculated, with reference to upturns and downturns in the level of help pursuant to the pandemic. A fourth source, “parents”, although taken into account in the SHARE questionnaire, was not included in our analysis due to the paucity of respondents who mentioned it. The question was phrased as follows: “*How often did the following people from outside your home help you to obtain necessities, compared to before the outbreak of Corona?”* listing as possible sources “*your own children*”, “*own parents*”, “*other relatives,*” and other non-relatives such as “*neighbours, friends, or colleagues*”. In addition, change in the frequency of such help due to the pandemic was investigated, the respondents being asked whether this support took place “*less often*”, “*about the same*” or “*more often*”, compared with the pre-pandemic period. A similar question was asked during the Wave 2-COVID-19 period, except that the term “compared to before the outbreak of the Corona” was replaced with the term “compared to the first wave of the pandemic”.

As for formal home care, the SHARE respondents during the Wave 1-COVID-19 period were asked whether they had regularly received formal home care before the pandemic broke out and whether they faced difficulties in obtaining the formal home care they needed afterwards, through the following question: “*Since the outbreak of Corona, did you face more difficulties in getting the amount of home care that you need*?” with “*Yes*” and “*No*” as possible answers. A similar question was asked during the Wave 2-COVID-19 period, through the following question: “*During the last three months, did you face difficulties in getting the amount of home care that you need?*” with “*Yes*” and “*No*” as possible answers. As no other questions about formal home care provision were asked in the survey, this variable was adopted as a proxy for formal home care, it being assumed that the expression “home care” captures the role of care services provided to community-dwelling older people in the countries involved in the study.

**Fig. 1:**
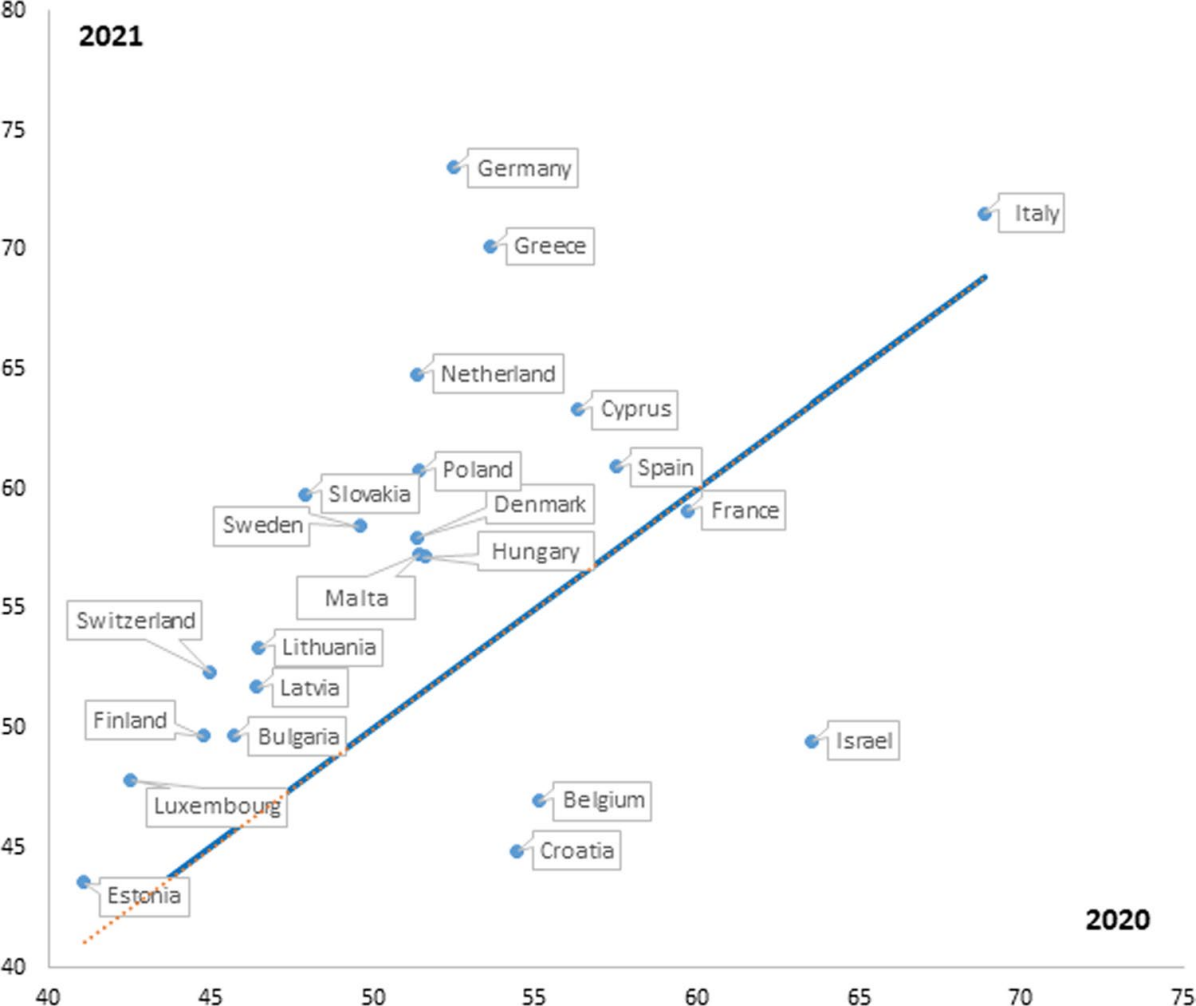
Value of Stringency Index in European countries in 2020 and 2021

In order to capture the overall impact of the different containment measures adopted by national governments to tackle the pandemic, we used the Stringency Index (SI) as calculated by the Oxford COVID-19 Government Response Tracker (OxCGRT) (for details about this tool refer to the project website: https://www.bsg.ox.ac.uk/research/covid-19-government-response-tracker). The SI records the strictness of containment policies that primarily restrict people’s behaviour, calculated using 9 ordinal indicators, 8 of which referring to containment and closure policies (school closing; workplace closing; cancel public events; restrictions on gathering size; close public transport; stay-at-home requirements; restrictions on internal movement; and restrictions on international travels), one reflecting the level of public information campaign adopted. Its value can range between 0 (meaning no restrictions) and 100 (indicating the highest level of restrictions). For 2020, the SI of each country was calculated as an average of the daily value of the SI recorded for all days between 1 February 2020 (start of the SI time series) and the ending date of the SHARE Wave 1-COVID-19 data collection (which was slightly different across countries, thus leading to a total period ranging between 178 and 196 days, depending upon the country). A similar approach was applied also to calculate the SI for 2021, in order to ensure comparability. Figure 1 illustrates the situation in the two considered years. Countries whose SI value is located under the bisecting line reported in 2021 a lower SI compared to 2020 (i.e. a relaxation of restrictions), the opposite being true for those located above the bisector.

In general, it can be observed that in most of the investigated countries (with few exceptions: Belgium, Croatia and Israel), containment policies became stricter in the first semester of 2021 compared to the first semester of 2020 (as reflected by the fact that most of them are located above the bisecting line).

### Analytical strategy

To identify country groups, a hierarchical cluster analysis methodology was used. The aim of this analysis was to detect, within the 23 EU Member States (including Israel) for which data were available, the presence of groups of cases that are both similar (i.e. presenting “maximum similarity”) within each group and, at the same time, as different as possible from the other groups (i.e. reflecting the “highest diversity” between clusters). To this end, the complete-linkage (or “furthest-neighbour”) method was used and the clusters were created by adding, in each step, the nearest case to all others already present in the specific group. The Squared Euclidean distance between cases was used to give a progressively greater weight to cases that are beyond a defined distance. Two indicators were used for this analysis: (a) one for informal care, represented by the average value obtained from the “difference between those reporting more and those reporting less practical help” calculated for each of the three sources of informal help considered (children, other relatives, and non-relatives such as neighbours, friends, etc.); and (b) one for formal home care, constituted by the “share of respondents reporting more difficulties in receiving home care from formal service providers”. At the end of the analysis, a final-cluster solution was obtained for 2020 and for 2021, and a further hierarchical cluster analysis was performed, in which clusters 2020 and clusters 2021 were used as indicators. This second step may be defined as a sort of “clustering of clusters”.

## Results

### Formal and informal home care provision during the Pandemic wave in the first half of 2021

**Fig. 2:**
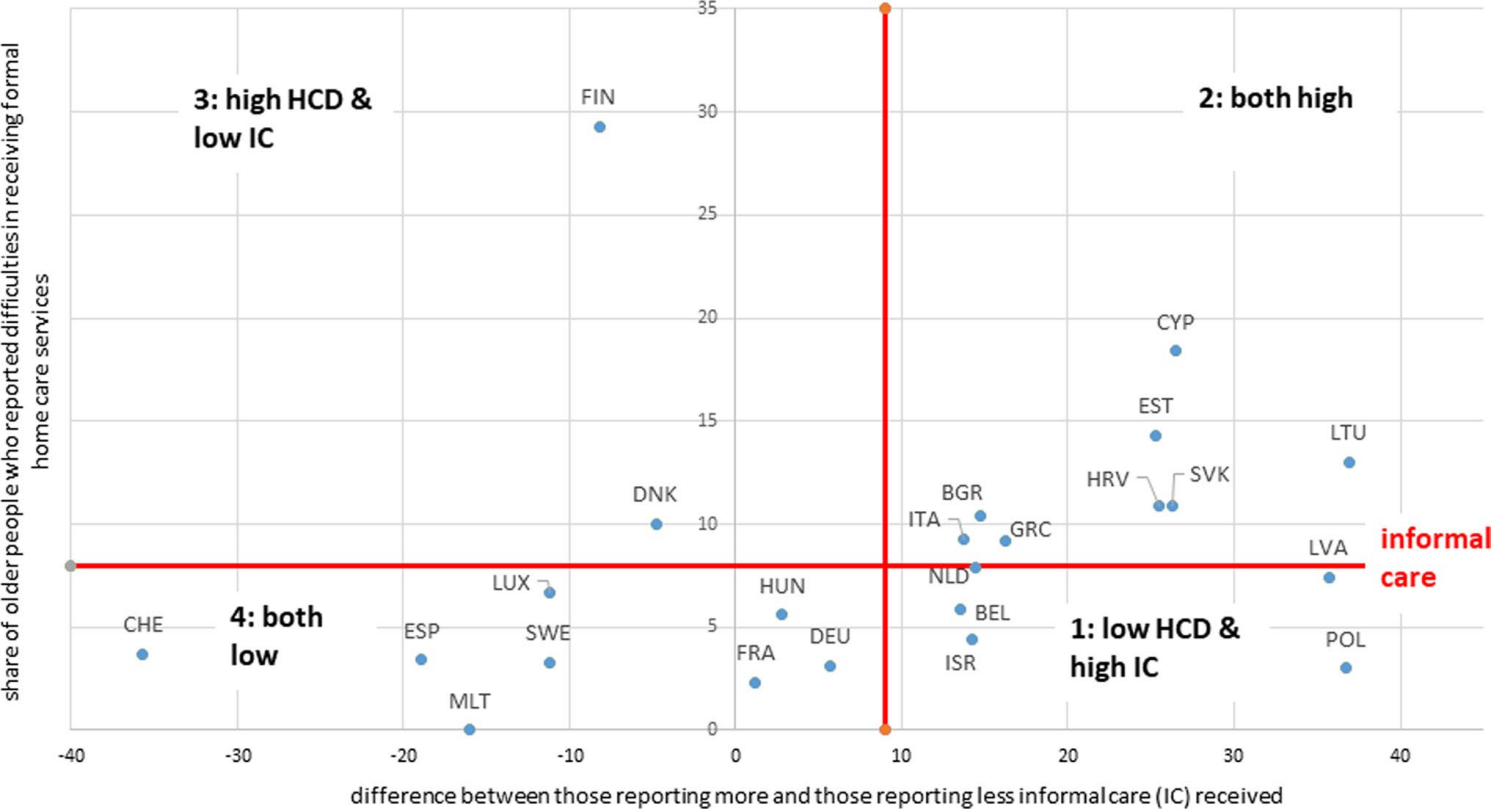
Difference between those reporting more and those reporting less informal care (IC) received share of older people who reported difficulties in receiving formal home care services in 2021. *BEL* Belgium, *BGR* Bulgaria, *HRV* Croatia, *CYP* Cyprus, *DNK* Denmark, *ESK* Estonia, *FIN* Finland, *FRA* France, *DEU* Germany, *GRC* Greece, *HUN* Hungary, *ISR* Israel, *ITA* Italy, *LVA* Latvia, *LTU* Lithuania, *LUX* Luxembourg, *MLT* Malta, *NLD* Netherlands, *POL* Poland, *SVK* Slovakia, *ESP* Spain, *SWE* Sweden, *CHE* Switzerland

During the pandemic wave that took place in the first half of 2021, the share of older people who reported difficulties in receiving formal home care services (vertical axis in Fig. 2) was rather low across countries, with the only remarkable exceptions of Finland (where almost one respondent out of three experienced this type of problem) and of Cyprus (where however this share did not reach 20% of respondents).

The pattern of informal care provision (horizontal axis) differed instead quite substantially across countries. This ranged between a group of Eastern European countries in which the provision of informal care increased strongly, by + 25% or more, compared to the first wave of the pandemic (most of them characterised also by a moderate level—i.e. + 10–15%—of respondents reporting difficulties in formal home care provision), to a large group of Continental and Mediterranean countries in which this increase was only minor (i.e. between 0 and + 15%), to a smaller group in which informal care provision decreased slightly (i.e. up to − 20%), up to the extreme case of Switzerland, which experienced a dramatic drop of almost − 40% in informal care.

### Comparison between the first (2020) and the second (2021) pandemic waves

**Fig. 3:**
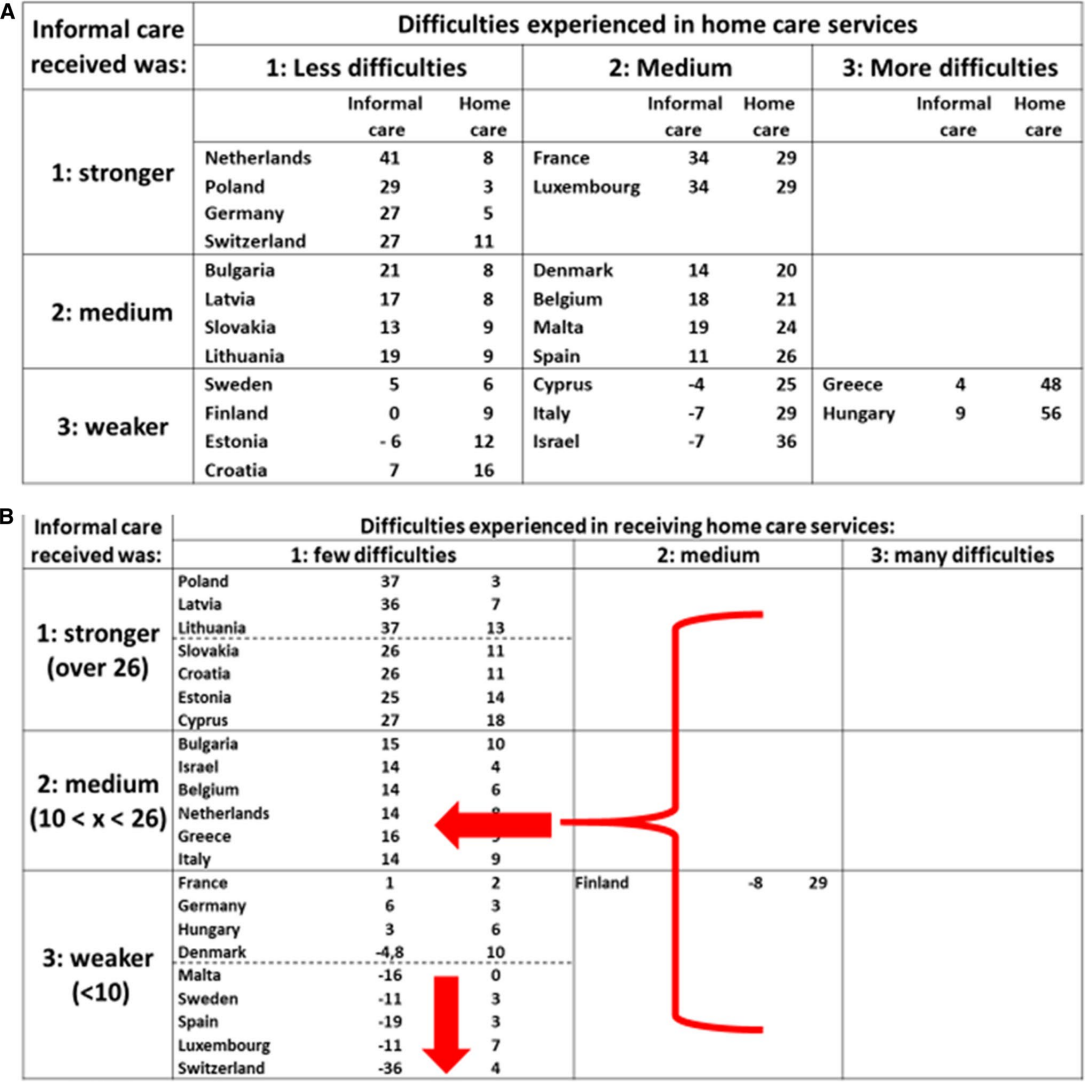
Change in informal and formal care received by older people during the first (2020) and second (2021) pandemic waves

When we compare the situation in 2021 with the one characterising the outbreak of the pandemic in 2020 (Fig. 3a, b, in which for comparability reasons cells reflect the same ranges of intervals for both years), we can observe two major changes between the two periods. The first concerns the disappearance of almost all countries (with the already mentioned exception of Finland) from the right hand columns “medium” and “more difficulties”, referring to the problems experienced by respondents in receiving formal home care services. This cross-nationally quite uniform change may possibly reflect the ability of formal home care systems to adjust over time to the challenges posed by the pandemic, by reactivating the formal home care provision disrupted during the first wave, which found care providers unprepared.

The second major trend to be observed concerns the substantial worsening of informal care provision in a number of countries (Luxembourg, Malta, Spain, Sweden, and especially Switzerland), to a level which was not reached during the first wave (thus potentially making them a sort of new cluster of countries). This phenomenon may be associated, on the one hand, to a weakening of the informal care network in these countries, possibly weary of supporting the cumulating impact of the COVID-19 pandemic waves over a longer period of time, to some extent also in substitution of formal home care provision (respondents of three of these five countries reported medium difficulties in receiving formal home care during the first wave, and four of them a medium or strong level of informal care). On the other hand, some of these countries (and especially Sweden and Switzerland) have notoriously adopted a rather “permissive” (i.e. non-restrictive) approach in terms of containment policies for longer periods of the COVID-19 pandemic, which may contribute to explain why informal care has dropped in these countries.

The two trends described above can be also visualised in Additional file 1: Fig. S1 (with regard to formal home care provision) and in Additional file 2: Fig. S2 (for informal care), by comparing values reported in 2021 (horizontal axis) and the percentage change occurred between 2021 and 2020 (vertical axis).

The values used to build the figures above are also reported in Table 1, which ranks countries in terms of the variation occurred for informal care provision between 2020 and 2021, thus contributing to better understand the two trends reported above. The data reported in the table show, on the one hand, the polarization occurring in terms of informal care provision, with several countries (Estonia, Cyprus, Italy, and Israel *in primis*) reporting a remarkable strengthening of this source of care over time, compared to the even more numerous group of countries (led by Switzerland, Luxembourg, Malta, and France) characterised by a noteworthy drop for this form of support.

On the other hand, the table highlights the evident improvement in terms of formal home care provision, with very few exceptions (Finland being, as already mentioned, the most remarkable one). What this table also allows to underline is that, for some countries, the two trends go in the same direction (either positive, as in Israel, Italy and Greece, or negative, as in Finland), in other countries they follow an opposite course (this being true especially for Luxembourg, Malta, France, and Spain).

By means of a cluster analysis, carried out by using the positions in the cells occupied by countries in the matrix matching the changes occurred between 2020 and 2021 in the provision of formal and informal home care (see Fig. 3), five groups of relatively homogenous countries could be found (see Table 2).

The first group includes primarily Eastern European countries (plus Belgium and the Netherlands) characterised by a rather stable position in the upper left corner of the matrix (cells 1 and 4 in Table 3). Cluster 2 concerns countries reporting a remarkable improvement especially in terms of informal care provision (thus moving from cells 7 and 8 upwards to cell 1), while cluster 3 includes Mediterranean countries improving in both areas (as reflected by their shift upwards and to the right from cells 8 and 9 to cell 4). The fourth group refers to countries especially from Continental Europe, reporting a strong drop in terms of informal care provision, counterbalanced by a substantial improvement in formal home care provision (thus “falling” down and to the left from cells 1, 2 and 5–7). Finally, the fifth and last cluster includes countries characterised by a stably weak and further weakening informal care provision (which keeps them in the bottom row of cells 7, 8 or 9), while remaining quite different in terms of formal home care provision.

### Correlations between the Stringency Index and changes in formal and informal home care provision

One aspect we tried to control for was also the possible existence of a relationship between the intensity of policy measures adopted to contain the COVID-19 pandemic (calculated using the Stringency Index, or SI, as previously described in the methodology section), on the one hand, and the change in the level of provision of both informal and formal home care in each of the investigated periods, on the other hand. Table 4 reports the correlations among the three investigated indicators, considered for each of the two analysed years 2020 and 2021 as well as in terms of the difference in values between the 2 years. Focussing our analysis on correlations between different types of indicators (thus excluding those highlighted in yellow, given the high level of correlation when you deal with the difference between values referring to various years of the same indicator), and limiting our comments only to the statistically significant findings, we observe that the level of difficulties reported in receiving formal home care in 2020 across countries is strongly correlated with the level of SI calculated for the same year. A second result suggests that the level of difficulties emerged in formal home care delivery in 2021 is somehow correlated to the situation of informal care provision of the previous year. (The stronger was informal care in 2020, the smaller the difficulties reported in formal home care in 2021.) Third, we note that the stronger the difference between the levels of informal care provided in 2020 and 2021 (difference that, as we know from Fig. 3, consists primarily in a reduction of informal care support in 2021 compared to the previous year), the more intensive are the difficulties reported for delivering formal home care in 2021. Finally, it is worthwhile to underline that there seem to be no overall correlation between the differences over time of the three indicators (cells in red), thus suggesting that, apart from the findings reported above, the three phenomena do not appear to be intrinsically connected.

**Table 1:**
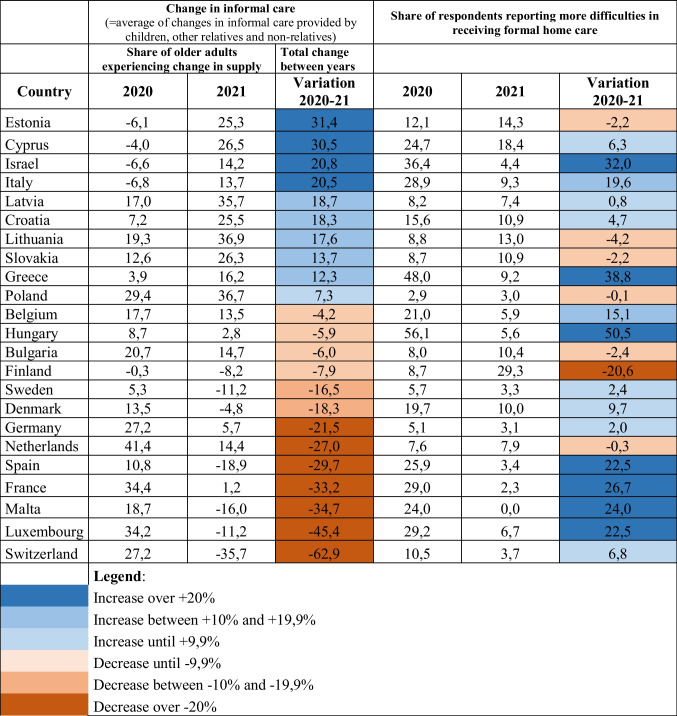
Countries by change in level of informal and formal care provision in 2020 and 2021 (ranked by level of variation 2020-21 in informal care provision)

**Table 2:**
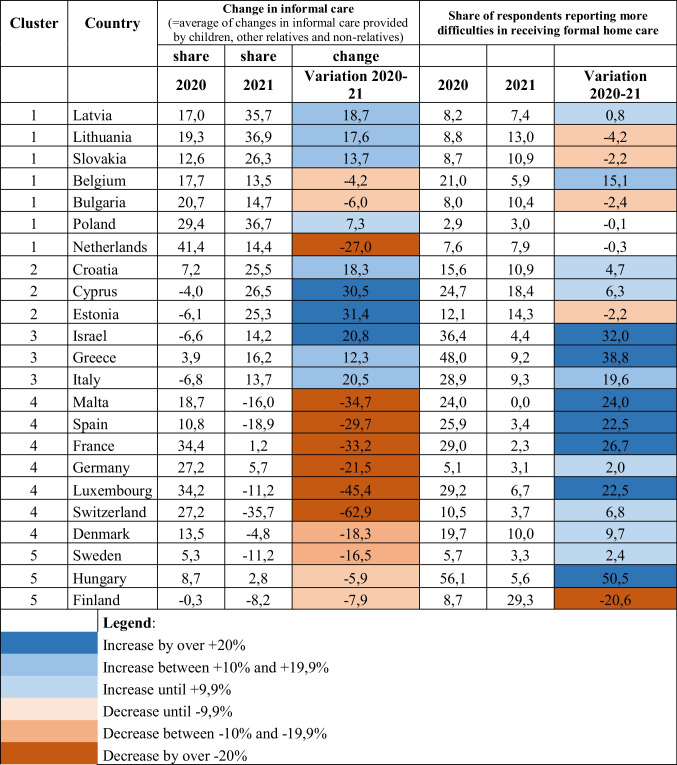
Countries by change in level of informal and formal care provision in 2020 and 2021 (ranked by level of variation 2020-21 in informal care provision)

**Table 3:**
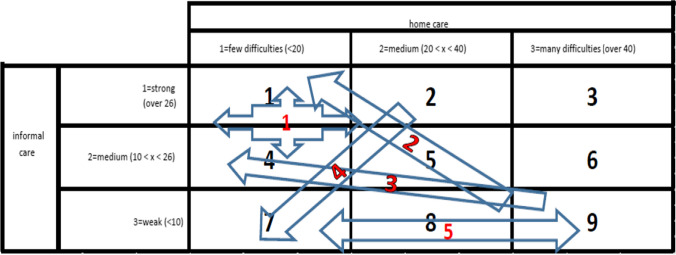
Clusters of countries by direction of change in level of informal and formal care provision in 2020 and 2021

**Table 4:** Matrix of correlation between the Stringency Index, informal care cluster, and the formal home care cluster variables, 2020 and 2021

	Stringency Index 2020	Stringency Index 2021	Stringency Index difference	Informal care 2020	Informal care 2021	Informal care difference	Difficulties in formal home care 2020	Difficulties in home care 2021	Formal Home care difference
Stringency Index 2020	1								
Stringency Index 2021		1							
Stringency Index difference			1						
Informal care 2020	− 0.2979 (0.1674)	0.0741 (0.7368)	0.3111 (0.1485)	1					
Informal care 2021	0.0403 (0.8551)	− 0.0003 (0.9989)	− 0.0586 (0.7907)	− 0.1652 (0.4513)	1				
Informal care difference	0.1865 (0.3941)	− 0.0424 (0.8478)	− 0.2101 (0.336)			1			
Difficulties in formal Home care 2020		0.1403 (0.5231)	− 0.2038 (0.3509)	− 0.3011 (0.1627)	− 0.1706 (0.4363)	0.0331 (0.881)	1		
Difficulties in formal home care 2021	− 0.2893 (0.1806)	− 0.2121 (0.3312)	0.0104 (0.9626)		0.2499 (0.2502)		− 0.1524 (0.4875)	1	
Formal Home care difference		0.1989 (0.363)	− 0.1801 (0.411)	− 0.0783 (0.7226)	− 0.2413 (0.2674)	− 0.1416 (0.5194)			1

Correlation level appears in the first line, significance level appears in the parenthesis

## Discussion

This paper has highlighted, to our knowledge for the first time in such a comparative and interconnected way, a series of trends in formal and informal home care provision to frail older people during the first two years of the COVID-19 pandemic. A first phenomenon, captured in Fig. 1, concerns the recognition that, across Europe, overall containment policies became stricter in the first semester of 2021 compared to the first half of 2020. These was partly a result of creation of task forces in most OECD countries to respond to the crisis, which included experts from the national, local or facility level. Moreover, about half of the countries have created specific task forces for long-term care services. Although much of their attention devoted to long-term care facilities, they also devoted resources and activities to the needs of frail older adults in the community. Indeed, we have shown that the provision of formal home care services to older people improved in the investigated period, as in 2021 the share of those who reported difficulties in receiving this kind of support dropped significantly compared to the previous year. According to a comprehensive report on the impact, policy responses and challenges of OECD countries to pandemic (Rocard et al. 2021), countries implemented measures to ensure community care continuity, by improving coordination between community long-term care, primary care and hospitals and increased coordination with social care services. Several OECD countries expanded the use of telehealth services to allow remote consultations between older adults and their families and the healthcare and social sector. Countries also increased the development of services and interventions using technology and especially digital solutions to facilitate contacts between care recipients and care providers as well as to promote peer support.

By contrast, informal care provision patterns experienced a growing polarization, with some countries continuing in reporting a strong support from this source, and others moving instead towards a situation of extreme reduction in the help coming from informal networks.

These two diverging trends characterising formal and informal home care may be due to a series of different circumstances partly differing from country to country. Moreover, it may be related to pre-pandemic informal care patterns. For example, most of the care for older adults in Eastern European countries fell on the shoulders of family members even before the pandemic, since formal home care in these countries was limited even before the pandemic.

However, also in light of what is emerging from the literature (Bagaria et al. 2022; Daly 2022; Dubois et al. 2022; Rocard et al. 2021; Tsopra et al. 2021), they seem to reflect two major phenomena. With regard to formal home care provision, we observe cross-nationally its capacity to adjust over time to the challenges posed by the pandemic, by reorganising home care services after the disruptions experienced initially at the outbreak of the pandemic (one major exception being Finland, probably in connection to the major reform affecting its long-term care system, Ylinen et al. 2021).

As for informal care provision, its contraction in a number of countries, sometimes to very low levels compared to pre-pandemic times, has been also found in other studies. This may be associated to a possible weakening of informal networks, worn out of the cumulating impact of the COVID-19 pandemic over a longer period of time, which obliged informal cares to jump in and replace the partially suspended or reduced formal care provision (Kilaberia et al. 2020; Budnick et al., 2021; Lorenz-Dant et al. 2021; Bergmann and Wagner 2021). The decline in informal care in Western European countries may be related to caregiver concerns due to employment, reducing their labour market attachment, or leaving the job market entirely, and subsequently sacrificing income and due increased psychological strain and worsened their financial situation (Eurocarers 2021; Rodrigues 2021). It is also important to note that there are differences between findings of various studies, apparently related to the study population, the period studied, and methodology. For example, while we found improvement in formal care in Germany, other surveys indicated the use of formal services decreased, especially in the case of respite care and support groups, but also in home visiting services and counselling by mobile care services.

The clustering of countries according to their prevailing behaviour with regard to the two components underlines that traditional welfare or care regime groupings (Ferrera 1996; Bettio and Platenga 2004) may still be relevant to interpret the impact of the recent pandemic. They indeed facilitate our understanding of the why, for instance, so many Eastern European countries have been consistently reporting few disruptions in terms of home care provision (given their little developed offer in this regard) and at the same time a stable informal support, being the latter the primary source of help for older people with long-term care needs in this area of the continent (Ariaans et al. 2021). The same framework seems to be in place and underpin the improvement reported between 2020 and 2021 by several Mediterranean countries (with the exception of Spain), especially—but not only—in terms of informal care provision, which was heavily hit by the containment measures adopted to contrast the first pandemic wave.

Less clear seems instead the contribution of traditional welfare and care regime theories in understanding the strong drop in informal care provision experienced by several non-Mediterranean countries reporting, at the same time, a substantial improvement in home care provision. These may be due to pandemic-intrinsic effects which, however, do not seem to be fully captured by the analysis of the possible correlations existing between the change in overall containment policies (as reflected by the Stringency Index), on the one hand, and the trends characterising the provision of both informal and home care. Future studies, considering also data based on the successive waves of the COVID-19 pandemic, will be helpful to better understand whether this dramatic drop in informal care provision represents a long-to-last legacy of the pandemic, or only a transient phenomenon.

## Limitations

This study has several limitations that should be considered. First, since two cross-sectional surveys in two points of time were compared, we cannot determine causality. Processes and measures that might been taken in between the surveys, such as formal home care policy reforms or economic incentives (to both formal and informal caregivers), in addition to the effects of the pandemic itself, may have well impacted the provision of both formal and informal home care and therefore the results of our analysis. Secondly, we compared between countries without differentiating between regions within the same country, although the pandemic has shown sometimes remarkable regional variations. Similarly, demographic and epidemiological characteristics of the investigated countries were not taken into account by the here presented analysis. Thirdly, the apparently surprising lack of association observed between the SI and the supply of informal and formal home care may well be due to the fact that none of the nine items on which the SI itself is based refers to care-related restrictions. Therefore, it is certainly possible that the SI-score is unable to capture trends occurring in the (elder) care sector. However, to our knowledge no better index is available to follow COVID-19-related policy responses, and this has to be considered a limitation of this study. Also, fourth, it should be noted that it is not known whether the Stringency Index (SI) had an effect depending on the indicator and countries. Such information could have been useful in better capturing the dynamics behind the changes in both formal and informal home care provision. Thirdly, the SHARE data used for this study are based on self-reporting and may therefore reflect primarily a subjective perspective and recall bias, rather than capture more macro-level, objective phenomena. This aspect should also be borne in mind in conjunction with the fact that we are not aware of the developmental process of the SHARE-COVID survey questionnaire, including its items addressing care-related issues. Therefore, we adopted the question on “home care” as a proxy for formal home care, in order to conduct a comparison with questions asked in previous waves.

Despite these limitations, this study presents novel findings on how the pandemic has affected the provision of care for older adults, both formally and informally, across a large set of countries. These findings can serve as a basis for the development of evidence-based recommendations that can inform future care policies at the national level. Since most of the frail older adults live in their homes in the community and since informal carers, especially family members, represent the vast majority of caregivers of them, policymakers should draw from these insights to ensuring that community services will better prepared to face future emergencies, reform current care systems, and implement more sustainable models for older adults living in the community. Such models should include a comprehensive and timely data systems, a regular standardised reporting and monitoring systems, ensuring access to information about guidelines, guidance and procedures, as well as means to supply better emotionally and financially support to informal carers. While this study represents a first step in this direction, further research is urgently needed to expand on these findings by examining the formal and informal home care dimensions within a more comprehensive conceptual framework.

### Supplementary Information

Below is the link to the electronic supplementary material.

**Supplementary file 1**. Fig. S1: Share of older people reporting difficulties in receiving formal home care in 2021 and change in share of older people reporting difficulties in receiving formal home care services between 2021 and 2020 (in % to previous year). Note: BEL=Belgium; BGR=Bulgaria; HRV=Croatia; CYP=Cyprus; DNK=Denmark; ESK=Estonia; FIN=Finland; FRA=France; DEU=Germany; GRC=Greece; HUN=Hungary; ISR=Israel; ITA=Italy; LVA=Latvia; LTU= Lithuania; LUX= Luxembourg; MLT= Malta; NLD= Netherlands; POL= Poland; SVK= Slovakia; ESP=Spain; SWE=Sweden; CHE=Switzerland.

**Supplementary file 2**. Fig. S2: Share of older people reporting difficulties in receiving informal home care in 2021 and change in share of older people reporting difficulties in receiving formal home care services between 2021 and 2020 (in % to previous year). Note: BEL=Belgium; BGR=Bulgaria; HRV=Croatia; CYP=Cyprus; DNK=Denmark; ESK=Estonia; FIN=Finland; FRA=France; DEU=Germany; GRC=Greece; HUN=Hungary; ISR=Israel; ITA=Italy; LVA=Latvia; LTU= Lithuania; LUX= Luxembourg; MLT= Malta; NLD= Netherlands; POL= Poland; SVK= Slovakia; ESP=Spain; SWE=Sweden; CHE=Switzerland.
