# Supplementary figures and images for: Comparing the cross-national impact of the COVID-19 pandemic on care received by community-dwelling older adults in 2020 and 2021: restoring formal home care versus polarizing informal care?
Source: Eur J Ageing. 2024 Jan 24;21(1):6. doi: 10.1007/s10433-024-00800-6 (PMC10808427; doi:10.1007/s10433-024-00800-6)

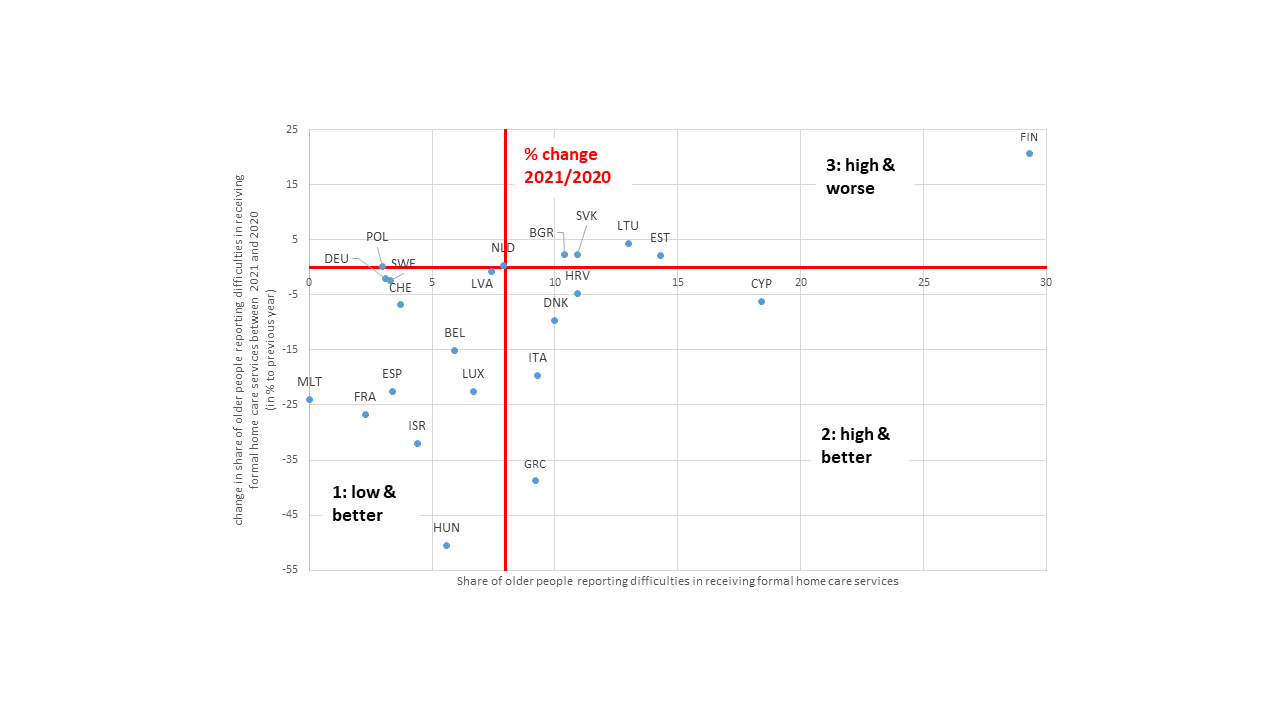

Supplement: Supplementary file 1 — Fig. S1: Share of older people reporting difficulties in receiving formal home care in 2021 and change in share of older people reporting difficulties in receiving formal home care services between 2021 and 2020 (in % to previous year). Note: BEL=Belgium; BGR=Bulgaria; HRV=Croatia; CYP=Cyprus; DNK=Denmark; ESK=Estonia; FIN=Finland; FRA=France; DEU=Germany; GRC=Greece; HUN=Hungary; ISR=Israel; ITA=Italy; LVA=Latvia; LTU= Lithuania; LUX= Luxembourg; MLT= Malta; NLD= Netherlands; POL= Poland; SVK= Slovakia; ESP=Spain; SWE=Sweden; CHE=Switzerland. [file 10433_2024_800_MOESM1_ESM.tif]

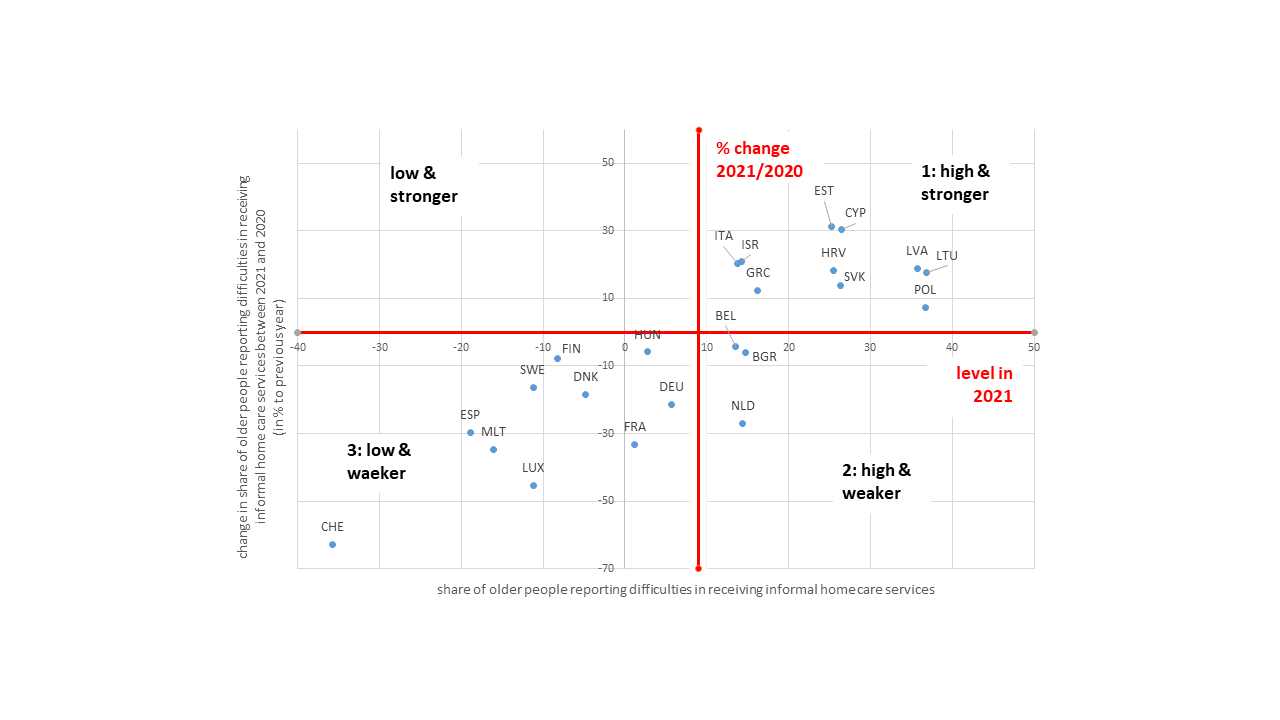

Supplement: Supplementary file 2 — Fig. S2: Share of older people reporting difficulties in receiving informal home care in 2021 and change in share of older people reporting difficulties in receiving formal home care services between 2021 and 2020 (in % to previous year). Note: BEL=Belgium; BGR=Bulgaria; HRV=Croatia; CYP=Cyprus; DNK=Denmark; ESK=Estonia; FIN=Finland; FRA=France; DEU=Germany; GRC=Greece; HUN=Hungary; ISR=Israel; ITA=Italy; LVA=Latvia; LTU= Lithuania; LUX= Luxembourg; MLT= Malta; NLD= Netherlands; POL= Poland; SVK= Slovakia; ESP=Spain; SWE=Sweden; CHE=Switzerland. [file 10433_2024_800_MOESM2_ESM.tif]
